# Early and late outcomes after open ascending aortic surgery: 47-year experience in a single centre

**DOI:** 10.1007/s00380-017-1075-3

**Published:** 2017-11-23

**Authors:** Emily Pan, Ville Kytö, Timo Savunen, Jarmo Gunn

**Affiliations:** 1 Heart Center, Turku University Hospital and University of Turku, Turku, Finland; 2 Center of Applied and Preventive Cardiovascular Medicine, University of Turku, Turku, Finland

**Keywords:** Aorta, Aortic arch, Aortic dissection, Aortic surgery, Aortic root, Outcomes

## Abstract

The aims of the study are to describe the long-term survival of patients undergoing primary open ascending aortic surgery and to portray the evolution of aortic surgery during six decades in a single centre. Included were all 614 patients who underwent primary ascending aortic surgery in 1968–2014 at one Nordic university hospital. Patients were identified and data were collected from patient records and surgical logs. Mortality data were acquired from the national registry. Median follow-up was 11.2 years using reverse Kaplan–Meier method. Overall 30-day survival was 91.2% and for 30-day survivor rates were 86.9, 77.6, 52.1, 38.3 and 26.7% at 5, 10, 20, 30 and 40 years. There was no significant difference in long-term survival for 30-day survivors (*p* = 0.105) between patients treated emergently for dissection/rupture and electively (mainly ascending aortic aneurysms). On Cox regression era of surgery (*p* = 0.006), increasing age (*p* < 0.001) and indication (*p* < 0.001) were predictors of 30-day mortality. Arch involvement indicated twofold risk (HR 2.09, *p* = 0.05) compared to non-arch involved. Only increasing age (*p* < 0.001) predicted long-term mortality. There was a sixfold risk of 30-day mortality in the earliest era compared to the latest (*p* = 0.03). After the early postoperative phase following ascending aortic surgery, the surgical indication and urgency of the index operation have no significant impact on long-term survival. The very long term survival after ascending aortic surgery is excellent for 30-day survivors and improved through the era. Surgical treatment has improved and perioperative mortality has decreased significantly in 47 years.

## Introduction

Thoracic aortic diseases (TADs) have increased steadily in recent years. The most common diseases are aneurysms, dissections or ruptures. The annual incidence of thoracic aortic aneurysms and dissections has previously been estimated at 16.3/100,000 for males and 9.1/100,000 for females, with an increasing trend in recent years [1]. Previous studies have established age, atherosclerosis, hypertension, the bicuspid aortic valve, connective tissue diseases such as Marfan syndrome or Ehlers–Danlos syndrome, previous cardiac surgery and family history as risk factors for thoracic aortic aneurysms (TAAs) and ascending aortic dissections (AADs) or ruptures. It is known that the size of the aorta correlates with the risk of complications [2] and the current guideline-based threshold for surgical intervention is aortic diameter ≥ 5.5 cm, regardless of aetiology [4]. Once dissected, the mortality for those surviving until surgical theatre has been reported at 25.1% [3]. If ruptured, the overall mortality reaches over 94% [5].

Cooley and DeBakey performed the first successful ascending aortic resection in 1951 using an allograft and on cardiopulmonary bypass [6]. The surgical techniques have since developed over the years. Many studies have shown that both postoperative in-hospital and later mortality for ascending aortic dissections (type A) have significantly decreased in recent years [7–12]. Nevertheless, the follow-up periods have mostly been short and very few clinical studies have been conducted to evaluate the long-term outcome of TADs for those who survived the critical initial 30-day period.

Due to the relatively low prevalence of TADs, and the relatively late adoption and development of operative treatment, long-term survival after TAD surgery still remains unclear. The purpose of this study is to investigate short- and long-term mortality after surgical treatment of ascending thoracic aortic diseases and to depict the evolution of ascending aortic surgery over the course of six decades.

## Materials and methods

This is a retrospective study consisting of all 614 consecutive patients who underwent primary ascending aortic surgery in a single centre, Turku University Hospital, Southwest Finland, between 1968 and 2014. Patients who underwent ascending aortic and/or aortic arch surgery with or without descending aortic surgery were included.

For estimation of postoperative mortality, patients who deceased during operation were excluded, following recommendations for reporting results after valvular surgery by the guidelines of the American Association for Thoracic Surgery, The Society of Thoracic Surgeons, and the European Association for Cardio-Thoracic Surgery [13].

The current geographical catchment area of TAD operations in Turku university hospital was established in 1984; therefore, we included only patients from 1984 to 2014 for population incidence calculations. The incidence concerns only surgically treated TAD patients aged ≥ 18 years. Incidence was standardized using the 2000 US Standard population with direct method.

Baseline data were collected retrospectively from patient records and surgical logs. One patient (1/614) was lost during the follow-up. The mortality and population data were provided by the Finnish national registry, Statistics Finland.

Due to the long follow-up, timing of surgery was classified into 4 eras: 1968–1980 (as there were only 6 patients during 1968–1970), 1981–1990, 1991–2000 and 2001–2014. Age was classified into ≤ 40 (14.7% of patients), 41–50 (13.9%), 51–60 (27.5%), 61–70 (29.4%) and ≥ 70 (14.4%) years. Four categories were created according to the surgical approach that was used or intended to use on the patient: modified Bentall procedure which involves replacement of the ascending aorta and aortic root with a composite tube graft and valve prosthesis (biological or mechanical) and coronary reimplantation as described by Inberg et al. [14]; modified Bentall procedure with concomitant coronary bypass (CABG) and/or mitral valve replacement (MVR) was classified as modified Bentall + CABG/MVR; valve-sparing David or Yacoub procedure with coronary reimplantation; isolated interposition with a tube graft of the ascending aorta and lastly hemiarch if the inner curvature of the aortic arch was replaced or total arch if distal anastomosis lied distal to the left subclavian artery with arch vessel reimplantation either separately or as islands. The indication for surgery was classified into those who had acute or chronic ascending aortic dissection, aortic rupture or intramural haematoma (i.e. aortic emergencies), and those who had other elective indications (namely TAA with or without aortic insufficiency). Survival analysis included only patients alive after operation and reoperations consisted of either resternotomies within the same hospital period or reoperations requiring a resternotomy after hospital discharge.

The study was approved by the institutional review board.

### Statistics

Chi-squared tests were conducted to depict interactions between age, sex, era of surgery, operation numbers, indication for operations and surgical approaches. Kaplan–Meier survival estimates were used for the long-term survival for 30-day survivors. The survival was also explored by indication and by decade of the surgery. Log-rank test was used to test the differences between the groups. Cox proportional hazards regression modelling was conducted to identify predictors of the short- and long-term mortalities. *p* values < 0.05 were inferred statistically significant. All statistical analyses were performed with SPSS IBM Statistics version 22.0 (IBM, Armonk, NY, USA).

### The evolution of surgical techniques

In the 1970s, a modified Bentall technique, as described by Inberg et al. [14], was adopted in Turku university hospital for reconstruction of the root and ascending aorta and thereafter was used as the preferred approach for root replacement in both acute and elective cases. In cases of a competent valve and minimal root involvement, an isolated supracoronary replacement of the ascending aorta was used. Hemiarch and total arch replacements were adopted in the 2000s for pathologies of the ascending aorta and were rarely used in earlier years. Valve-sparing (David) techniques for root surgery were adopted in 2009 and used thereafter for suitable elective cases.

Until the late 1990s, cannulation was performed with direct cannulation of a femoral artery for acute aortic dissection. Thereafter, the most common arterial cannulation was switched to cannulation through an 8-mm end to side vascular prosthesis to the femoral artery. In salvage cases, however, direct cannulation was still employed for rapid access. For elective cases, arterial cannulation was located at the lesser curvature of the arch for ascending aortic surgery and femoral artery for arch repair. Venous drainage was achieved either through the femoral vein or right atrium. Direct selective cannulation of supra-aortic vessels was used for arch repair.

Hypothermia for elective cases of ascending aortic surgery was at 30–34 centigrade and until the mid-2000s, the ascending aortic surgery was performed on-clamp. Total circulatory arrest and deep hypothermia (20–24 degrees centigrade) with open distal anastomosis was adopted along with hemiarch repair during 2000s. Deep hypothermia and circulatory arrest with selective antegrade cerebral perfusion was used for arch repair during acute setting.

## Results

The total study population consisted of 614 patients who had undergone an ascending aortic operation. The median follow-up was 11.8 years using reverse Kaplan–Meier method (range 0–46.8) for the entire cohort, and the overall mean age at the time of surgery was 56.0 ± 14.4 years (range 18–84). As shown by Table 1, the average age increased significantly (*p* < 0.001) for patients undergoing ascending aortic operations over the decades: less than 40 years old decreased from 48.0 to 10.0%, from the 1960s to the 2010s, respectively, while at the same time the proportion of over 70 years old increased from 0.0 to 25.4%. Of all patients, 33.7% were treated for aortic dissections or ruptures, the rest 66.3% had mainly elective aneurysms. The number of males who underwent TAD surgery was fourfold compared to females (*n* = 491, 80% vs. *n* = 123, 20%), but females tended to have more dissections and ruptures than males (*p* = 0.023).

**Table 1:** Patient characteristics stratified by time of ascending aortic surgery

Variable	1968–1980	1981–1990	1991–2000	2001–2010	2011–2014	Total	*p* value
Sex							0.259
Male	36 (72.0%)	72 (83.7%)	112 (84.8%)	169 (78.2%)	102 (78.5%)	491 (80.0%)	
Female	14 (28.0%)	14 (16.3%)	20 (15.2%)	47 (21.8%)	28 (21.5%)	123 (20.0%)	
Age							< 0.001
≤ 40	24 (48.0%)	25 (29.1%)	18 (13.6%)	9 (4.2%)	13 (10.0%)	89 (14.5%)	
41–50	7 (14.0%)	16 (18.6%)	28 (21.2%)	25 (11.6%)	9 (6.9%)	85 (13.8%)	
51–60	13 (26.0%)	28 (32.6%)	40 (30.3%)	57 (26.4%)	32 (24.6%)	170 (27.7%)	
61–70	6 (12.0%)	17 (19.8%)	39 (29.5%)	77 (35.6%)	43 (33.1%)	182 (29.6%)	
≥ 70	0 (0.0%)	0 (0.0%)	7 (5.3%)	48 (22.2%)	33 (25.4%)	88 (14.3%)	
Operation number	49 (8.0%)	87 (14.2%)	132 (21.5%)	216 (35.2%)	130 (21.2%)	614 (100%)	
Indication^a^							0.003
Dissection/rupture	8 (16.3%)	34 (38.4%)	55 (42.3%)	77 (35.6%)	343(25.4%)	207 (33.7%)	
Others (mainly aneurysm)	41 (83.7%)	53 (61.6%)	75 (57.7%)	139 (64.4%)	97 (74.6%)	404 (66.3%)	
Surgical procedure							< 0.001
Bentall	22 (44.0%)	57 (66.3%)	70 (53.0%)	144 (66.7%)	74 (56.9%)	367 (59.8%)	
David	0 (0.0%)	0 (0.0%)	0 (0.0%)	5 (2.3%)	14 (73.7%)	19 (3.1%)	
Interposition	26 (52.0%)	21 (24.4%)	36 (29.5%)	20 (9.3%)	19 (14.6%)	122 (19.9%)	
Arch repair	1 (2.0%)	3 (3.5%)	6 (4.5%)	24 (11.1%)	10 (7.7%)	44 (7.2%)	
Bentall + CABG/MVR	1 (2.0%)	5 (5.8%)	20 (15.2%)	23 (10.6%)	13 (10.0)	62 (10.1%)	
Resternotomy^b^	0	5	1	13	1	20	

^a^ *n* = 614

^b^ During the same period of treatment

**Fig. 1:**
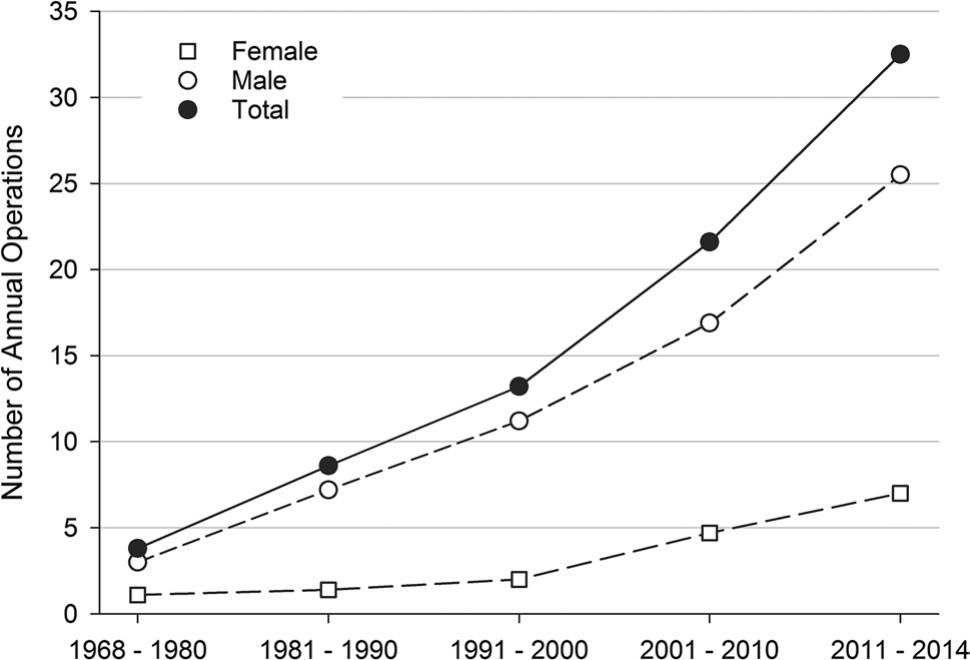
Annual number of operated ascending aortic diseases per decade

**Fig. 2:**
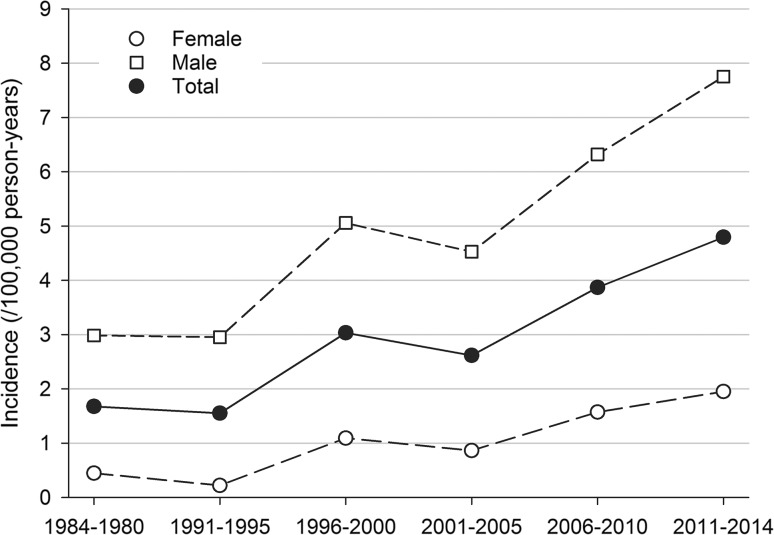
Average sex-specific incidence rate per decade per hundred thousand inhabitants for operated ascending aortic diseases (1984–2014)

The number of surgically treated TADs increased steadily from an average of 3.8 operations per year in the 1960s and 1970s to 32.5 operations per year in the 2010s (Fig. 1). During 1984–2014, the incidence of surgically treated TADs was 0.96 cases per 100,000 per year for females, 4.72 cases per 100,000 per year for males and overall 2.78 per 100,000 inhabitants per year. The incidence increased significantly from 1984 to 2014 in both sexes, from 1.68/100,000/year in 1984–1990 to 4.80/100,000/year in 2011–2014. The increase was more prominent for females than for males (0.45–1.95 per 100,000 vs. 2.98–7.76 per 100,000) (Fig. 2). The Bentall procedure has remained the most frequent surgical approach, at an average of 59.8% of all surgeries. In the 1968–1980 supracoronary replacement of the ascending aorta with a tube graft (i.e. interposition) was the most employed approach of all (49.0%) but dropped notably towards the twenty-first century (14.6%).

### Postoperative survival

Among all those who survived the index procedure, the postoperative survival was 86.8, 79.5, 70.3, 47.1, 34.6 and 24.2% at 1, 5, 10, 20, 30 and 40 years after operation, respectively. In total, 223 patients (39.6%) died during follow-up from 1968 to 2014. Postoperative 30-day mortality was 9.2% overall, 18.3% (OR 1.75, CI 1.27–2.41) for emergent dissections or ruptures and 5.5% (OR 0.45, CI 0.35–0.60) for other indications (*p* < 0.001). For all patients who survived up to hospital admission, the perioperative mortality was 7.5% and the 30-day mortality rate was 16.0%. The long-term survival for 30-day survivors was 86.9, 77.6, 52.1, 38.3 and 26.7% at 5, 10, 20, 30 and 40 years. The mean survival after 30 days was 10.4 years (median 7.7, SD ± 9.5, interquartile range 9.6–11.2).

**Fig. 3:**
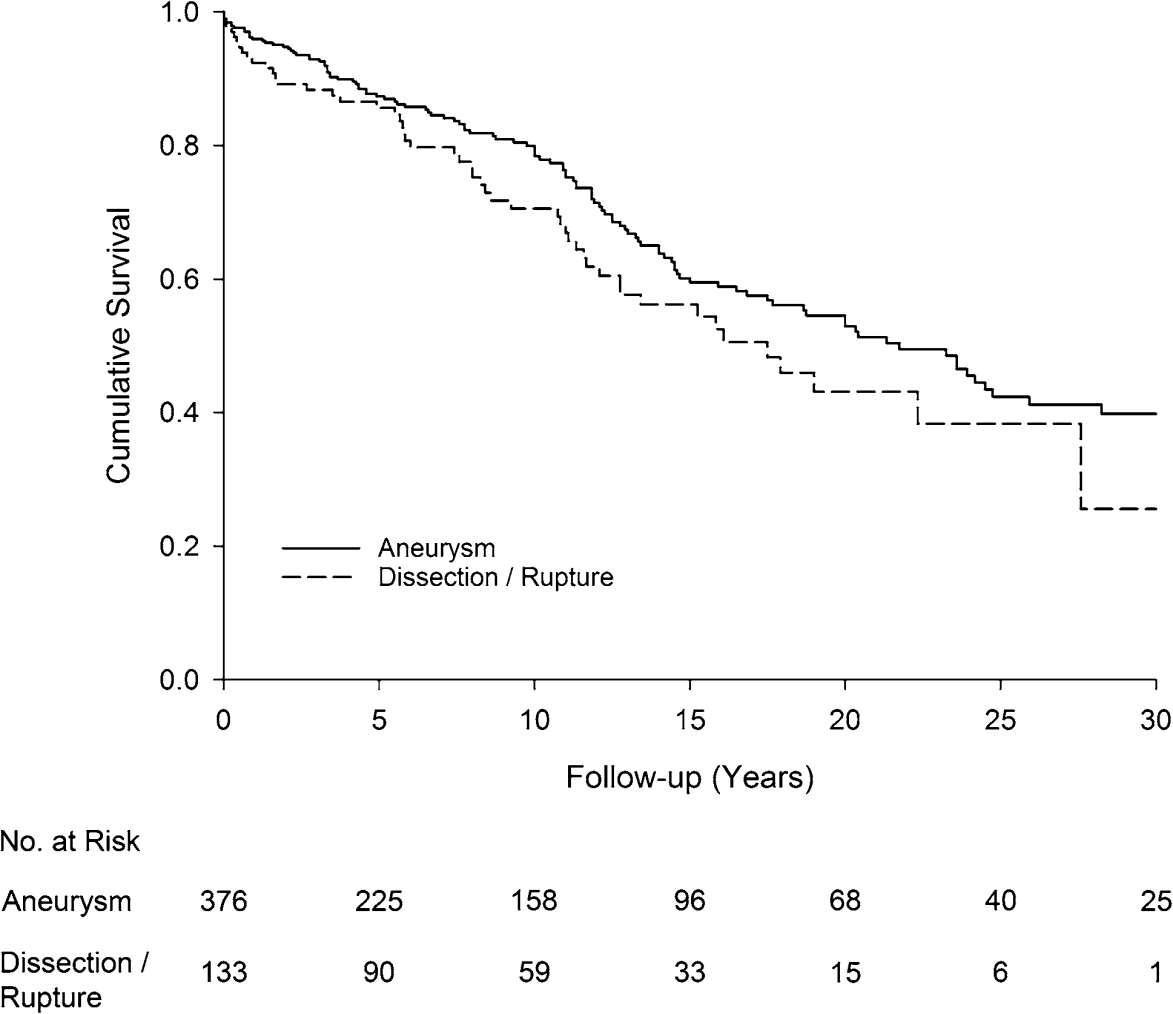
Kaplan–Meier survival curve for 30-day survivors stratified by surgical indication: acute dissection/rupture vs. elective surgery (mainly aneurysms). There were no statistical difference in long-term survival between the groups (*p* = 0.096). Numbers at risk are indicated

The Kaplan–Meier analysis with log-rank test showed a significant difference in 30-day mortality (*p* < 0.001) between patients treated emergently for dissection/rupture and electively for mainly aneurysms. There was no difference (*p* = 0.096) in long-term survival for those who survived the first 30 days (Fig. 3). On a multivariable Cox proportional hazard model, increasing age and the indication for surgery predicted 30-day mortality. There was a trend towards a greater mortality with arch involvement (*p* = 0.056). There was a sixfold hazard of 30-day mortality in the earliest era of TAD surgery compared to the latest era (1968–1980 vs. 2010–2014, HR 6.0, *p* = 0.002). For long-term survival for 30-day survivors, increasing age was the only significant predictor for mortality (*p* < 0.001) (Table 2).

**Table 2:** Variables of 30-day mortality and long-term mortality for 30-day survivors after ascending aortic surgery

	30-day mortality				Long-term mortality for 30-day survivors			
	Univariate		Multivariate		Univariate		Multivariate	
	HR (95,0% CI)	*p* value	HR (95,0% CI)	*p* value	HR (95,0% CI)	*p* value	HR (95,0% CI)	*p* value
Sex (male vs. female)	0.83 (0.43–1.62)	0.586	0.97 (0.49–1.92)	0.93	1.13 (0.74–1.61)	0.548	1.23 (0.81–1.86)	0.327
Age (per one year)	1.06 (1.03–1.08)	<0.001	1.05 (1.02–1.08)	0.002	1.04 (1.03–1.05)	<0.000	1.05 (1.03–1.06)	0.000
Indication^a^	3.58 (2.07–6.21)	<0.001	3.95 (2.16–7.23)	< 0.001	1.31 (0.94–1.83)	0.107	1.16 (0.80–1.68)	0.442
Era (vs. 2011–2014)		0.001		0.002				
1968–1980	1.32 (0.41–4.28)	0.646	6.00 (1.60–22.53)	0.008	0.96 (0.39–2.40)	0.842	2.16 (0.83–5.59)	0.113
1981–1990	0.35 (0.76–1.63)	0.332	0.89 (0.23–3.46)	0.870	1.02 (0.43–2.42)	0.931	1.58 (0.66–3.82)	0.306
1991–2000	0.35 (0.09–1.28)	0.112	0.37 (0.10–1.41)	0.147	1.21 (0.52–2.82)	0.967	1.37 (0.58–3.21)	0472
2001–2010	2.34 (1.12–4.91)	0.019	1.90 (0.90–4.01)	0.094	1.21 (0.52–2.80)	0.656	1.12 (0.48–2.63)	0.786
2011–2014						0.654		
Surgical approach (in addition to only ascending aortic procedure)								
+Arch	3.79 (1.90–7.56)	< 0.001	2.09 (0.98–4.45)	0.056	0.92 (0.43–1.96)	0.829	0.83 (0.38–1.78)	0.625
+Aortic valve	1.11 (0.58–2.11)	0.757	1.56 (0.76–3.22)	0.225	0.80 (0.58–1.12)	0.190	1.11 (0.67–1.48)	0.976
+CABG/MVR^b^	1.62 (0.76–3.44)	0.210	2.18 (0.97–4.93)	0.060	1.34 (0.82–2.19)	0.242	1.11 (0.66–1.85)	0.697

^a^ Indication = dissection/rupture vs. elective surgery (mainly aneurysms)

^b^ Coronary artery bypass grafting/mitral valve replacement

### Reoperations

The cumulative incidence of postoperative resternotomy during the same hospital period was 3.2% (*n* = 18). Cumulated incidence of reoperation after hospital discharge was 2.3% (*n* = 13), of which four patients (25%) died during reoperation. Freedom from reoperation was 98.1, 98.1, 95.6, 94.0 and 86.2% at 5, 10, 20, 30 and 40 years. The indication for reoperations was thoracic aortic aneurysm (*n* = 3), abdominal aortic aneurysm (*n* = 2), aortic dissection (*n* = 2), chronic pericarditis (*n* = 2), arrhythmia (*n* = 2), thoracic aortic rupture (*n* = 1), abdominal aortic rupture (*n* = 1), mechanical valve infection (*n* = 1), aortic valve stenosis (*n* = 1), mitral valve regurgitation (*n* = 1), coronary artery disease (*n* = 1) and coronary artery aneurysm (*n* = 1).

## Discussion

To our knowledge, this is the longest follow-up of patients treated surgically for ascending aortic diseases, starting from the very first ascending aortic operation performed in Finland in 1968 continuously until 2014, 47 years in total with 100% completeness of follow-up. We describe six decades of aortic surgery and depict trends in surgical approaches in both short-term and very long-term survival.

In previous studies, the late survival for hospital survivors has been reported lower for acute dissections [9, 10, 15, 16]. In a Swedish nationwide population-based study the survival for > 30 days from operation for both aneurysms and dissections was 77, 57 and 43% at 5, 10, and 15 years, respectively [1]. Our total follow-up was more than 30 years longer and the survival rate was higher. In addition, the perioperative and initial 30-day mortality of aortic dissections are also comparable with a previous study [17].

Interestingly, our data showed no difference in long-term survival for 30-day survivors, regardless of aetiology. Our results suggest that surgical indication and approach have little effect on long-term prognosis; if patients survive the critical 30-day postoperative period, long-term survival for ascending aortic dissections or ruptures appears to be as good as for ascending aortic aneurysms.

Postoperative resternotomies during the same hospital admission were surprisingly infrequent and the rate of reoperations after discharge was even lower in our data, also indicating excellent surgical result. Previous studies have reported reoperation rate to be from 7.8 to 23.2% and that reoperation rates were not associated with preoperative aortic regurgitation severity, surgical technique nor any other factors for that matter [1, 10, 18]. Reoperations are technically challenging due to tight adhesions and the mortality still remains high especially for distal re-dos [19]. It can be speculated that actual indications for reintervention on the aorta are hitherto unclear and represent subjective institutional and surgeon-dependent thresholds for surgery, but further studies on subject are warranted. However, our late reoperation rates should be interpreted with caution as possible reinterventions performed at other institutions cannot be completely accounted for even if treatment of ascending aortic pathologies is centralized within geographic areas in Finland. Nevertheless, it is unlikely that the number of reoperations unaccounted for in these data would be dramatic.

We found a steady increase in incidence of TAD operations over past six decades for both sexes. Similar trends have been described by several previous studies as well [1, 7, 20]. This study, like most previous studies, describes only the incidence of operated TADs and did not include patients who were treated conservatively or who died prior to hospital admission. Thus, the true incidence of TADs is expected to be higher than reported here, or previously, due to its predominantly silent character and high pre-hospital mortality during an acute event. In fact, according to a previous study, 95% of TADs were reported asymptomatic before an acute event, such as dissection or rupture [21]. Improved imaging techniques and their better availability in addition to better awareness and understanding of the disease have improved the diagnostics of TADs, but whether the true incidence of TAD has increased lately remains unclear.

Very long outcomes of patients with ascending aortic diseases and especially ascending aortic aneurysms have not been extensively described. Previous studies have mostly been either longer follow-ups with few cases from a single centre or short follow-ups with large data from centres specialized in aortic diseases. Our data consist of an extensively collected all-comers cohort from a tertiary centre that covers unselected patients in need of aortic surgery in Southwest Finland. The current study describes real-world outcomes, which are possibly better applicable to the majority of western cardiac surgical units than results from highly specialized aortic centres.

The immediate main goal during an aortic catastrophe is to perform necessary operational care while ensuring survival. In smaller cardiothoracic centres, this is achieved by minimizing the complexity of the procedure. The more complex the procedure, such as elephant trunks in hybrid techniques after initial ascending aortic repair, the longer the patient has to be under perfusion and anaesthesia, leading to a higher rate of postoperative ischemic events and early mortality. It is also imperative that the techniques used are replicable by all cardiac surgeons performing on-call duties. Some studies have suggested that more complex operations may lead to better long-term survival and reduction in rates of reoperations [22–24]. Our study showed an extremely low rate of reoperations and an excellent long-term survival after the initial 30 days.

## Limitations

The major limitations of this single-centre study are associated with the retrospective nature of the database. Although data were checked directly from patient files, incompleteness is still a possibility. During decade-long follow-up, the development of surgical techniques, engineering, devices, and the improvement of perioperative and postoperative management have been radical, but this is also one the main focus of this study, i.e. the evolution of surgical management of ascending aortic pathologies.

## Conclusions

Our study shows that a relatively small cardiothoracic surgery centre can produce comparable results with specialized expert centres in both emergent and elective ascending aortic surgery. This puts into question the often-suggested high-volume requirements for quality ascending aortic surgery. Our results also suggest that an initially straightforward and simple surgical approach yields good long-term prognosis indicating that a KISS (keep it short and simple) approach is justified in ascending aortic surgery.
